# Transgenic tomato line expressing modified *Bacillus thuringiensis cry1Ab* gene showing complete resistance to two lepidopteran pests

**DOI:** 10.1186/2193-1801-3-84

**Published:** 2014-02-12

**Authors:** Bhupendra Koul, Sugandha Srivastava, Indraneel Sanyal, Bhuminath Tripathi, Vinay Sharma, Devindra Vijay Amla

**Affiliations:** Plant Transgenic Lab, CSIR-National Botanical Research Institute, Rana Pratap Marg, P.O. Box 436, Lucknow, 226 001 India; Department of Microbiology, King George’s Medical University (KGMU), Lucknow, 226 003 India; Department of Botany, Guru Ghasidas Vishwavidyalaya, Bilaspur, 495 009 Chhattisgarh India; Department of Biosciences & Biotechnology, Banasthali Vidyapith, P.O. Banasthali, Tonk Road, Rajasthan, 304 022 India

**Keywords:** *Agrobacterium tumefaciens*, Bt-*cry1Ab*, Genetic transformation, Insect mortality, Tomato, Vegetative leaves

## Abstract

The modified truncated Bt-*cry1Ab* gene of *Bacillus thuringiensis* has been used for the development and selection of over expressing transgenic events in a commercially important variety of tomato (*Solanum lycopersicum* L.) by *Agrobacterium*-mediated leaf-disc transformation procedure. The integration and inheritance of *cry1Ab* gene in T_0_ transgenic plants and their progenies were determined by PCR, RT-PCR and Southern blot hybridization analysis. The toxin expression was monitored by double antibody sandwich enzyme-linked immunosorbent assay (DAS-ELISA). The transgenic line Ab25 E, expressing 0.47 ± 0.01% Cry1Ab toxin of total soluble protein (TSP) was finally selected in the T_4_ generation from the segregating population, showing 100% mortality to the second instar larvae of *H. armigera* and *S. litura* and minimal damages to leaves and fruits. Southern blot analysis data revealed single copy introgression of *cry1Ab* gene in highly-expressing Ab25 E transgenic line and expression of Cry1Ab toxin of molecular mass ~65 kDa was evident in Western blot analyses in transgenic plants of T_4_, T_5_ and T_6_ generation. Receptor binding assay performed with partially purified Cry1Ab protein from Ab25 E transgenic tomato line, confirmed efficient protein-protein interaction of Cry1Ab toxin with receptor(s) of both the insects. The higher level of Cry1Ab toxin (≈ 0.47 ± 0.01% TSP) did not affect the normal *in vitro* regeneration, plant development and fruit yield in this transgenic line. This high expressing Cry1Ab homozygous transgenic line can be a useful candidate in tomato breeding programmes for introgression of important agronomical traits.

**Electronic supplementary material:**

The online version of this article (doi:10.1186/2193-1801-3-84) contains supplementary material, which is available to authorized users.

## Background

Tomato (*Solanum lycopersicum* L.) is a major vegetable crop plant and extensively consumed either raw or cooked. Besides being a dietary source of antioxidants, vitamins, minerals and fiber it is also a model system for studies on fruit development and functional genomics (Klee and Giovannoni 2011). A wide range of microbial pathogens and insect pests are known to attack tomato, particularly polyphagous lepidopteran insect *Helicoverpa armigera*, the common fruit borer which primarily damages the fruit, while *Spodoptera litura* damages the leaves causing severe losses to the crop productivity. There is a possibility of developing stable insect-resistant tomato lines through the expression of insecticidal gene of *Bacillus thuringiensis* (Bt), as documented successfully in several crop plants like cotton, maize, soybean, rice, canola and potato (Sanahuja et al. 2011; Tabashnik et al. 2011). The application of Bt-toxins for insect pest resistance has emerged as a powerful tool, being chemically free, eco-friendly and highly specific against target insects due to the presence of specific receptors in the midgut, while being non-toxic to beneficial insects and vertebrates owing to the lack of the receptors for toxin interaction and binding (Pigott and Ellar 2007; Bravo et al. 2011). Incorporation of *cry1Ab* insecticidal crystal protein gene in large number of crop plants particularly rice, tomato, maize, sugarcane and cotton have shown considerable protection against different lepidopteran insects and significant enhancement in productivity (Ye et al. 2001; Kumar and Kumar 2004; Dutton et al. 2005; Arvinth et al. 2010; Khan et al. 2013).

The Cry1Ac toxin of *B. thuringiensis* has broader specificity than those of Cry1Aa and Cry1Ab and is primarily a class 1 and 3 aminopeptidase N (APN) binding protein whereas Cry1Aa and Cry1Ab are class 1 APN binding proteins (Pigott and Ellar 2007). Although Cry1Ac has been documented to be most effective toxin against *H. armigera* owing to its binding to different receptors in target insect but is ineffective against *S. litura* (Bravo et al. 2004; Purcell et al. 2004). It is also reported that the rate of pore formation by Cry1Ac is lower than that of Cry1Ab (Kato et al. 2006). The Cry1Ab toxin has been reported to interact by diverse modes in monomeric and oligomeric forms with brush border membrane vesicles of different lepidopteran insects for pore-formation (Zhang et al. 2005,2006; Kato et al. 2006; Padilla et al. 2006; Vachon et al. 2012; Pardo Lopez et al. 2013). Moreover, over-expression of Cry1Ac toxin in plants is a major constrain for being toxic to *in vitro* regeneration and development of transformed plant cells. Selection of transgenic events expressing high-levels of Cry1Ac toxin is still not a routine procedure (Diehn et al. 1996; De Rocher et al. 1998; Mehrotra et al. 2011; Rawat et al. 2011). Interestingly, *cry1Ab* gene shares significant homology with *cry1Ac* (Schnepf et al. 1998) which is also effective against large number of lepidopteran insects and is at the same time non-toxic during *in vitro* development of transgenic plants expressing high-levels of the toxin (Estela et al. 2004; Padilla et al. 2006; Pacheco et al. 2009). To overcome these problems, several modifications have been incorporated in the native *cry1Ac* and *cry1Ab* genes for stability of the transcripts including, elimination of polyadenylation sites, inclusion of plant-preferred codons and enhanced GC ratio (Perlak et al. 1990; De Rocher et al. 1998).

Considering these facts we have adopted the strategy of over-expressing the modified truncated version of *cry1Ab* gene for the selection of transgenic events over expressing Bt Cry1Ab toxin in a commercial tomato variety Pusa early dwarf (PED), which is grown round the year throughout North India. In this paper we have reported the selection of high expressing stable transgenic lines of tomato in T_4_ generation after extensive screening and selection of progenies starting with the promising T_0_ transgenic plants. Insect bioassay performed under laboratory conditions with detached leaves and fruits of transgenic line Ab25 E over expressing Cry1Ab toxin showed complete mortality of two polyphagous insect pests *H. armigera* and *S. litura*. The T_6_ generation of this transgenic tomato line, did not show any negative effect on plant growth, development and fruit yield compared to non transgenic control plants.

## Results

### *Agrobacterium-*mediated transformation and regeneration of transgenic tomato plants

**Figure 1:**
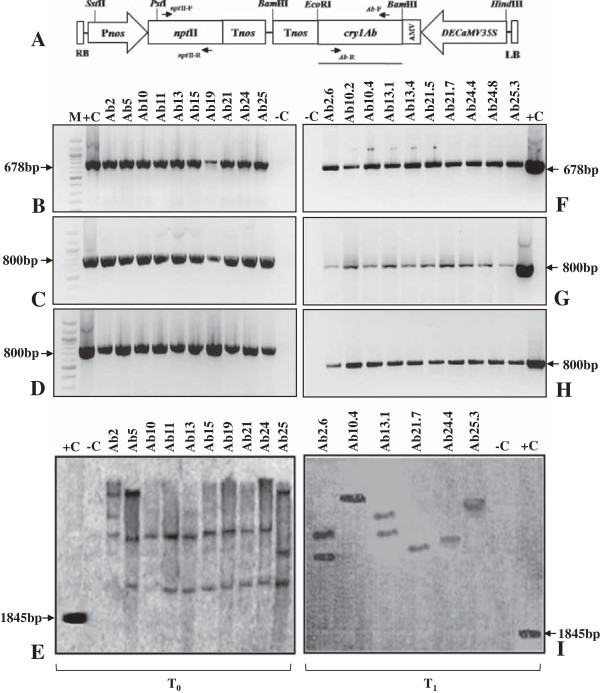
**Schematic diagram of the T-DNA region of binary vector pBIN200 and molecular characterization of T**_**0**_ **and T**_**1**_ **transgenic tomato plants. A** T-DNA region of pBIN200 harbouring *cry1Ab* gene driven by constitutive *DECaMV35S* promoter. Different PCR primers used in the present study are shown with arrow marks at their respective binding positions: *npt*II-F, *npt*II-R for *npt*II gene and *Ab*-F, *Ab*-R for *cry1Ab* gene amplification, respectively. Bold line represents 1845 bp *Bam*HI and *Eco*RI fragment of *cry1Ab* gene used for preparation of radiolabelled probe for Southern blot hybridization analysis in the present study. RB and LB: right and left border sequences, *npt*II: neomycin phosphotransferase gene, AMV: alfalfa mosaic virus 5′ UTR sequence, *cry1Ab*: modified truncated 1845 bp *cry1Ab* gene of *B. thuringiensis*, *DECaMV35S*: *CaMV35S* promoter with duplicated enhancer, P*nos*: *nos* promoter, T*nos*: *nos* terminator. PCR amplification of genomic DNA from transgenic tomato in T_0_ and T_1_ generations. **B**, **F** 678 bp amplification of *npt*II gene. **C**, **G** 800 bp amplification of *cry1Ab* gene. **D**, **H** RT-PCR analysis of T_0_ and T_1_ transgenic tomato showing 800 bp amplicon of *cry1Ab* gene transcripts, respectively, +C: truncated 1845 bp *cry1Ab* gene as positive control. –C: DNA from non-transformed plant as negative control. **E**, **I** Southern blot analysis of PCR positive T_0_ transgenic plants and randomly selected T_1_ transgenic plants. +C: 1845 bp fragment of truncated *cry1Ab* gene. –C: DNA from non-transformed control plant.

The excised and preconditioned vegetative leaf explants of commercial variety of tomato (PED) complementary to *Agrobacterium*-mediated transformation were used, for high frequency recovery of T_0_ transgenic plants along with effective management of escapes and development of chimeric transgenic plants. Leaf disc explants that were kept on MS basal medium for *in vitro* regeneration served as positive control, while those on kanamycin-supplemented medium served as the negative control. Every responding leaf disc on an average produced about 5.80 number of elongated shoots per explant in 96±2% responding explants on MS basal medium supplemented with optimal concentration of carbon source (3% maltose) and 2.5 mgl^-1^ BAP (Table 1). Two consecutive selection cycles on kanamycin-supplemented medium resulted into a maximum transformation frequency of 28.20%, thus reflecting the efficacy of the optimized tomato transformation and regeneration procedure. The comprehensive data on tomato transformation with binary vector pBIN200 harbouring modified and truncated 1845 bp *cry1Ab* gene (Figure 1A) and selection of T_0_ primary transformants from a series of experiments is summarized in Table 2. A total of 143 T_0_ transgenic tomato plants were developed and categorized into three groups on the basis of quantitative expression of Cry1Ab toxin. The first group comprising of 86 plants were expressing Cry1Ab toxin maximally up to 0.002% of TSP; second group of 32 plants expressing up to 0.02% of TSP, while the third group comprising of 25 plants expressing Cry1Ab toxin ranging from 0.02–0.13% of TSP and were considered as the promising group of T_0_ transgenic plants for further investigation to select the high expressing transgenic lines (Additional file 1: Table S1).

**Table 1:** **Effect of carbon source and cytokinins on shoot regeneration from vegetative leaf explants in Pusa Early Dwarf (PED) variety of tomato**

Carbon source (%)	PGR^1^(mg l^-1^)	Responding explants^2^(%)	Elongated shoots per responding explant
2% Sucrose	1.0 BAP	55.26^a^	2.70^ab^
2% Sucrose	2.5 BAP	57.00^a^	2.93^b^
2% Sucrose	1.0 ZET	56.10^a^	2.98^b^
2% Sucrose	2.5 ZET	49.26^a^	1.95^ab^
3% Maltose	1.0 BAP	83.73^b^	1.57^a^
3% Maltose	2.5 BAP	96.20^b^	5.80^c^
3% Sucrose	2.5 BAP	87.28^b^	4.75^c^
3% Maltose	1.0 ZET	60.00^a^	2.20^ab^
3% Maltose	2.5 ZET	92.30^b^	2.70^ab^

Duncan’s Multiple Range Test (DMRT) was performed to compare means and data having different letter within a column are significantly different (*P* < 0.05).

^1^Plant growth regulator.

^2^Percentage of regenerating explants on media supplement under trial.

BAP: 6-Benzyladenine, ZET: Zeatin.

**Table 2:** **Tomato transformation, selection on kanamycin supplemented medium and regeneration of transgenic plants**

	I^st^selection				II^nd^selection				
Experiment	No. of explants used (A )	No. of responding explants (B)	% Response (B/A)	No. of shoot buds produced	No. of explants inoculated (C)	No. of responding explants (D)	% Response (D/C)	Antibiotic resistant plants produced (E)	% Frequency of transformation (E/A)
1.	138	50	36.23	63	186	83	44.62	39	28.20
2.	104	49	47.11	60	65	39	60.00	22	21.15
3.	123	59	47.96	76	226	76	33.62	28	22.76
4.	194	124	63.91	103	319	142	44.51	54	27.83
	559^c^	282^c^	48.80^d^	302^c^	796^c^	340^c^	45.68^d^	143^c^	24.98^d^
+Control^a^	40	40	100	114	230	221	96.08	-	-
-Control^b^	52	8	15.3	0	-	-	-	-	-

^a^Control tomato leaf explants incubated on MS medium devoid of kanamycin.

^b^Control tomato leaf explants incubated on MS medium with kanamycin.

^c^Total number of explants/plants.

^d^Average value.

### Molecular characterization of the transgenic plants

Ten T_0_ transgenic tomato plants from the third group expressing higher level of Cry1Ab toxin, were selected and verified for the incorporation of *npt*II and *cry1Ab* genes by PCR amplification and RT-PCR for *cry1Ab* transcript. Results of PCR and RT-PCR assays performed with these transgenic plants have shown the amplification of expected amplicons of 678 bp and 800 bp for *npt*II and *cry1Ab* genes respectively (Figure 1B–D). Southern blot hybridization of these ten T_0_ over expressing transgenic plants with 1845 bp *cry1Ab* gene probe revealed the independent nature of transgenic events. Most of the T_0_ transgenic plants showed double copy insertion while few plants showed single copy insertion of the transgene (Figure 1E). The T_1_ seeds of all the 25 T_0_ transgenic plants were screened for segregation of *npt*II gene in T_1_ progeny. Results showed typical 3:1 Mendelian ratio for segregation of *nptII* gene and differential quantitative expression of Cry1Ab toxin with corresponding mortality of *H. armigera* and *S. litura* larvae (Additional file 1: Table S1). Eventually, five over expressing T_0_ transgenic plants designated as Ab10, Ab13, Ab21, Ab24 and Ab25 expressing Cry1Ab toxin >0.07% TSP along with one transgenic plant Ab2 showing low expression (0.02% of TSP) were selected for further investigation and analysis (Additional file 1: Table S1). Quantitative Cry1Ab transcript analysis in these T_0_ transgenic plants was performed by quantitative PCR using *β*-actin gene of tomato as the internal control. Interestingly, Ab10, Ab24 and Ab25 transgenic plants showed significantly high-level of *cry1Ab* transcript, which was about eight times higher than the low expressing transgenic plant Ab2 (Additional file 2: Figure S1).

Ten T_1_ transgenic plants designated as Ab2.6, Ab10.2, Ab10.4, Ab13.1, Ab13.4, Ab21.5, Ab21.7, Ab24.4, Ab24.8 and Ab25.3 of respective Ab2, Ab10, Ab13, Ab21, Ab24 and Ab25 T_0_ parents were selected and subjected to PCR analyses. Results of PCR assays for *npt*II and *cry1Ab* genes showed the expression of expected amplicons of 678 and 800 bp respectively (Figure 1F, G). RT-PCR analysis for *cry1Ab* gene in these T_1_ plants also showed the expression of expected amplicon of 800 bp and reconfirmed the stable inheritance of the transgene (Figure 1H). Five of these T_1_ transgenic plants # Ab10.4, Ab13.1, Ab21.7, Ab24.4 and Ab25.3 over-expressing Cry1Ab toxin along with Ab2.6 T_1_ transgenic plants were used for Southern blot hybridization and results revealed a single-copy insertion of *cry1Ab* gene in transgenic plants ID # Ab10.4, Ab21.7, Ab24.4 and Ab25.3 and two copies in Ab2.6 and Ab13.1 plants respectively (Figure 1I).

### Growth characteristics of transgenic plants

Constitutive expression of Cry1Ab toxin by *DECaMV35S* promoter did not influence any apparent abnormalities on plant phenotype and overall growth and development of all the T_0_ transgenic plants. In order to accomplish our aim of selecting a highly overexpressing transgenic line, we selected a single T_1_ transgenic plant Ab25.3 derived from Ab25 T_0_ parent on the basis of Bt-Cry1Ab protein content for further investigation. Five over expressing lines of Ab25.3 T_1_ parent designated as Ab25 A, Ab25 B, Ab25 C, Ab25 D and Ab25 E were identified and selected in T_4_ generation on the basis of consistent expression of Cry1Ab toxin and overall growth and yield of transgenic plants. The transgenic plants of Ab25 E line consistently showed maximum expression of Cry1Ab toxin and normal growth amongst the five overexpressing transgenic lines and selected for detail characterization. Compared to the wild type, only the onset of flowering in the transgenic lines was delayed. The onset of flowering occurred after 30 ± 1 days in the wild type plants whereas 34 ± 2 days in Ab25 E line. The average number of fruits and seeds per plant in the wild type were 14 ± 4 and 2.57 ± 0.10, while in Ab25 E line it was 12 ± 2 and 2.49 ± 0.10 respectively. The average plant dry weight of Ab25 E transgenic line (510 ± 11.23 g) was near to the wild type (552 ± 45.28 g), suggesting no apparent affect on plant growth parameters between the wild type and transgenic plant (Table 3). Some of the high expressing transgenic tomato plants did show reduction in overall growth and fruit yield and were discarded (data not shown).

**Table 3:** **Comparative assessment of different growth parameters in selected high expressing transgenic tomato plants of T**_**4**_ **generation**

Transgenic lines	Cry1Ab toxin (% of TSP)	^a^Onset of flowering (days after sowing)	^b^Average no. of fruits / plant	^c^No. of seeds / fruit / g	^d^Dry wt. (g)
Wild type	-	30 ± 1	14 ± 4	2.57 ± 0.10	552 ± 45.28
Ab25 A	0.37 ± 0.004	40 ± 3 (0.013)^*^	11 ± 1 (0.225)	2.48 ± 0.08 (0.016)	475 ± 11.52 (0.059)
Ab25 B	0.19 ± 0.004	36 ± 2 (0.009)	9 ± 2 (0.049)	2.5 ± 0.10 (0.426)	456 ± 5.78 (0.052)
Ab25 C	0.14 ± 0.004	38 ± 2 (0.005)	8 ± 3 (0.009)	2.54 ± 0.20 (0.655)	450 ± 3.33 (0.052)
Ab25 D	0.39 ± 0.002	36 ± 3 (0.035)	11 ± 2 (0.122)	2.45 ± 0.09 (0.002)	482 ± 6.25 (0.090)
Ab25 E	0.47 ± 0.003	34 ± 2 (0.020)	12 ± 2 (0.225)	2.49 ± 0.10 (0.300)	510 ± 11.23 (0.166)

^a^Average number of days for onset of flowering from seed sowing, in 20 transgenic tomato plants of each line.

^b^Average number of fruits per plant in 20 transgenic tomato plants of each line.

^c^Average number of seeds (per gram of fruit) in eight fruits of individual transgenic plant, in 20 plants of each line.

^d^Dry weight of 20 transgenic tomato plants of each line.

^*^Values in parenthesis indicate the probability associated with a student’s paired *t*-test.

When *P*<0.05 the difference of the individual parametric value to that of the value of the control was significant.

### Quantitative Cry1Ab expression and corresponding insect mortality

**Figure 2:**
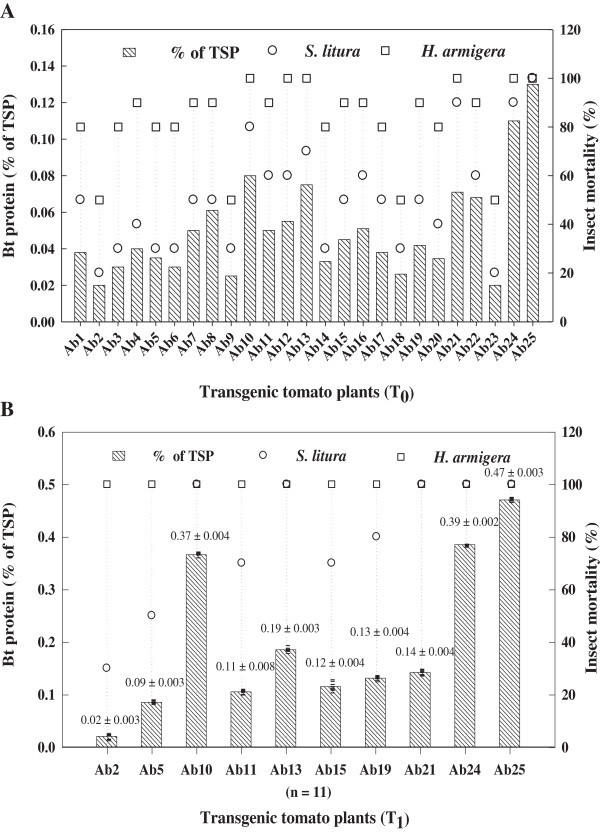
**Quantitative assessment of Bt-Cry1Ab toxin protein in different T**_**0**_ **plants and selected T**_**1**_ **population. A** and **B** by double antibody sandwich enzyme-linked immunosorbent assay (DAS-ELISA) as percentage of total soluble protein (TSP) (*striped bar*) and corresponding insect mortality of *H. armigera* (*open square*) and *S. litura* (*open circle*). Average quantity of Bt-Cry1Ab toxin in transgenic plants is shown as% TSP ± standard deviation on the top of histogram bars and also indicated by horizontal mark for individual transgenic plant and ‘n’ is the number of T_1_ transgenic plants.

The Cry1Ab toxin in T_0_ and T_1_ transgenic tomato plants was examined by DAS-ELISA and calculated as the percentage of Bt-toxin in total soluble protein (TSP). Cry1Ab toxin content in the third group of 25 independent T_0_ plants ranged between 0.02–0.13% of TSP and resistance bestowed against the fruit worm *H. armigera* and cut worm *S. litura* corresponding to the Cry1Ab level is shown in Figure 2A. The expression level of Cry1Ab toxin in T_1_ population was increased over their T_0_ parents. The T_1_ transgenic plants of the respective T_0_ parents Ab10, Ab24 and Ab25 showed 0.37 ± 0.004, 0.39 ± 0.002 and 0.47 ± 0.003% Cry1Ab toxin of TSP, respectively and resulted 100% mortality of *H. armigera* after 48 h feeding on the detached vegetative leaves (Figure 2B).

As expected the *cry1Ab* gene under *DECaMV35S* constitutive promoter was expressed in all the parts of T_0_ transgenic tomato # Ab25 plant and level of Cry1Ab toxin was in the order, flower > seed > sepal > raw pulp > raw pericarp > leaf > stem > root > ripe pericarp > ripe pulp (Additional file 3: Figure S2). A similar pattern of Cry 1Ab toxin expression was found in Ab25 E transgenic line in T_6_ generation (data not shown).

**Figure 3:**
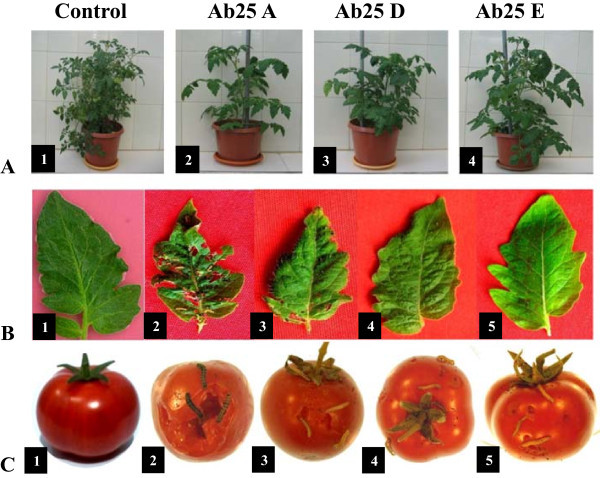
**Insect bioassay performed on Bt-transgenic tomato lines of T**_**4**_ **generation with 2nd instar larvae of** ***S. litura*** **and** ***H. armigera*** **. A** Tomato plants grown under contained glasshouse 1– non-transgenic plant as control, 2– Ab25 A, 3– Ab25 D and 4– Ab25 E transgenic plants. **B** Insect bioassay assay performed with *S. litura* on detached leaves of tomato plants documented after 48 h of feeding. 1– leaf of control plant without insect infestation, 2– leaf of control plant, 3– Ab25 A, 4– Ab25 D and 5– Ab25 E lines challenged with *S. litura* respectively showing significant damages to the control leaf compared to the transgenic leaves. **C** Insect assay performed with *H. armigera* on tomato fruits documented after 96 h of feeding, 1–non-transgenic tomato as control without insect infestation, 2–control tomato, 3–Ab25 A, 4–Ab25 D and 5–Ab25 E lines challenged with insect respectively showing significant damages to the control tomato compared to the transgenic tomato.

The T_4_ transgenic plants of Ab25A, Ab25B, Ab25C, Ab25D, Ab25E lines and control non transgenic plants developed under contained glass house conditions were subjected to extensive insect bioassay with both the insects. The detached leaves and fruits of the transgenic and control plants were fed to second instar larvae of *S. litura* and *H. armigera*, respectively and results obtained are shown Figure 3 and Additional file 4: Table S2. The leaves and fruits of transgenic lines # Ab25 A, Ab25 D and Ab25 E, challenged with larvae of *S. litura* and *H. armigera* showed complete protection against both the insects (Figure 3B-3 to 5, Figure 3C-3 to 5). Insect feeding on mature tomato fruits showed about 17.3% damage caused to the control fruits (Figure 3C-2), whereas, less than 2.0% damage was noticed in fruits of transgenic line Ab25 E (Figure 3C–5).

The percent leaf damages inflicted by *S. litura* on leaves of control plants after 48 h of feeding was 26.14 ± 3.91 while that to Ab25 A, Ab25 B, Ab25 C, Ab25 D, Ab25 E was found to be 0.39 ± 0.19, 3.48 ± 2.89, 3.75 ± 2.46, 0.25 ± 0.12 and 0.20 ± 0.16 respectively. Whereas, the percent damages incurred to the control fruits by *H. armigera* after 96 h of feeding was 17.30 ± 3.11 while that in Ab25 A, Ab25 B, Ab25 C, Ab25 D, Ab25 E transgenic plants was 2.96 ± 1.85, 3.54 ± 2.03, 4.70 ± 2.63, 2.72 ± 1.79 and 1.71 ± 1.18 respectively. The pictorial representation of percent leaf and fruit damages during insect bioassay are summarized in Additional file 5: Figure S3 and Additional file 6: Figure S4.

### Inheritance and expression of *cry1Ab* gene in transgenic plants

**Figure 4:**
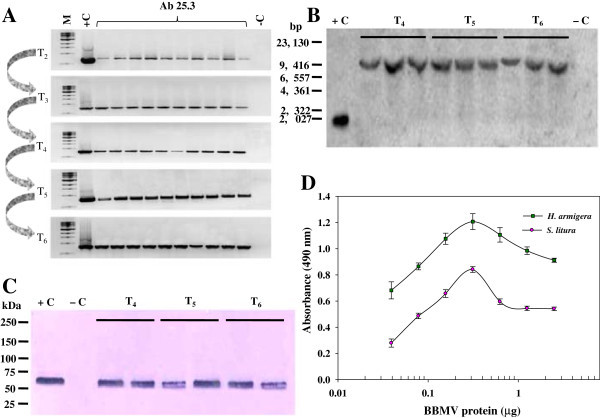
**Molecular characterizations of Bt-transgenic progenies and receptor binding assay. A** RT-PCR analysis of 10 selected transgenic tomato plants from T_2_ to T_6_ generation of T_1_ transgenic plant Ab25.3. M: 1kb ladder, *cry1Ab* amplicon size is 800 bp. **B** Southern blot hybridization of *Eco*RI digested DNA from transgenic tomato Ab25 E line, in T_4_, T_5_ and T_6_ generation, +C 1845 bp fragment of *cry1Ab* gene as positive control, –C: Genomic DNA from non-transformed tomato as negative control. **C** Western blot with protein extract from Ab25 E transgenic tomato line expressing *cry1Ab* gene in T_4_, T_5_ and T_6_ generations, +C: partially purified Bt-protein of ~65 kDa, –C as positive control: Total protein isolated from non-transformed tomato plant as negative control. **D** Binding assay of plant expressed Cry1Ab toxin protein with BBMV protein of *H. armigera* (■) and *S. litura* (○). The BBMV protein fraction (300 ng/100 μl) from both the insects were coated and shown in long scale while Cry1Ab toxin protein (125 ng/ 100 μl) was used for protein-protein interaction followed by immuno absorbance assay.

Inheritance of *cry1Ab* gene in the T_1_ population was analyzed by germination and screening of T_1_ seeds on kanamycin supplemented medium which revealed segregation according to Mendelian ratio 3:1 (resistant: susceptible, p ≤ 0.05, χ ^2^ = 3.841) for kanamycin tolerance (Additional file 1: Table S1). Results of semi-quantitative assessment for stable inheritance of the transgene in 10 randomly selected transgenic plants of Ab25.3 line from T_2_ to T_6_ generations are shown in Figure 4A. Southern blot analysis performed with T_4_, T_5_ and T_6_ progeny of Ab25 E transgenic line, showed a single band of approximately 10 kb in size, hybridizing with the *cry1Ab* gene probe (Figure 4B). No rearrangement of the *cry1Ab* gene was found in the T_6_ progeny, which indicates that *cry1Ab* transgene was faithfully transmitted into subsequent generations *via* selfing.

Results of Western blot analysis performed with cell-free protein extracts of T_4_, T_5_ and T_6_ progenies of Ab25 E transgenic line showed single band of molecular weight ~65 kDa hybridizing with the polyclonal Cry1Ab antibodies. The results on expression of toxin protein of ~65 kDa, as expected from 1845 bp *cry1Ab* gene in T_6_ stage of transgenic plants demonstrates the stable incorporation of *cry1Ab* gene at a unique locus into transgenic tomato line Ab25 E for stability and consistent over expression of the transgene (Figure 4C).

### Receptor binding assay

The binding efficiency of partially purified BBMV receptor fractions from 5th instar larvae of *H. armigera* and *S. litura* with partially purified recombinant Cry1Ab toxin expressed in transgenic tomato line Ab25 E was checked by DAS-ELISA. The results of the receptor binding assay performed with Cry1Ab toxin (125 ng/100 μl) showed concentration-dependent response and maximum absorbance of 1.2 and 0.8 with 300 ng/100 μl BBMV protein fractions of *H. armigera* and *S. litura* respectively. Results summarized in Figure 4D shows microgram (μg) quantities of BBMV protein on x-axis in logarithmic scale and corresponding absorbance on y-axis. The results of protein-protein interaction was 1.5 fold higher in *H. armigera* than *S. litura* indicating higher efficacy and abundant binding sites on the brush border epithelium membrane fraction of *H. armigera* compared to *S. litura*.

## Discussion

Both *H. armigera* and *S. litura* are serious pests to several important crops world over and particularly in India, affecting a large number of agricultural, forest and horticultural plants. These insects may damage over 45 to 48% of tomato plants resulting significant loss of tomato yield (up to 35 to 40%) in India. The expression of Bt-Cry toxins through transgenic technology in different crop plants for eco-friendly control of insect pests is a revolutionary advancement for enhancement of crop yield (Sanahuja et al. 2011; James 2012). Transgenic cotton and maize expressing recombinant Cry1Ac and Cry1Ab toxins were the first genetically transformed crops commercialized for resistance against the highly devastating Bollworm complex pest, including *H. armigera* and are currently being grown in over 120 million hectares all over the world (Perlak et al. 1990; Fearing et al. 1997; Perlak et al. 2001; Conner et al. 2003; James 2012).

The different Cry1A toxins (a, b, and c) share close resemblance in having three identical domains, structural homology and mode of action except for their binding efficiency onto the specific receptors and different epitopes of the same receptor such as cadherin-like proteins (CAD), alkaline phosphatase (ALP) and aminopeptidase (APN) (Pigott and Ellar 2007; Fortier et al. 2007). The Cry1Ac toxin is most effective against large number of lepidopteran insects due to efficient oligomerization and binding to these receptors particularly with alkaline phosphatase (Bravo et al. 2004; Upadhyay and Singh 2011). Whereas, Cry1Ab toxin is shown to bind rapidly with BT-R_1_ receptor involving the stimulation of G-protein adenylyl cyclase/PKA signaling pathway for destabilization of the midgut cytoskeleton, ion channel and pore formation via a different mode of action resulting to mortality of lepidopteran insects (Zhang et al. 2005,2006; Kato et al. 2006; Padilla et al. 2006). According to recent hypothesis, pore formation is attributed to interaction of Cry1A toxins first with ALP and APN to concentrate and resulting activated toxin to bind with CAD receptor involving three epitopes CR 7, CR 11 and CR 12 for Cry1Ac and only two epitopes CR11 and CR12 for Cry1Ab, therefore, reflecting differential order of toxicity Cry1Ac > Cry1Ab and minimal for Cry1Aa (Kato et al. 2006; Pardo Lopez et al. 2013).

Earlier studies on engineering of tomato expressing either *cry1Ab* or *cry1Ac* gene (Fischhoff et al. 1987; Delannay et al. 1989; Mandaokar et al. 2000; Kumar and Kumar 2004) have shown protection against either tomato fruit worm (*Heliothis zea*) and tobacco hornworm (*Manduca sexta*) or tomato fruit borer (*Helicoverpa armigera*). However, selection of the transgenic tomato line Ab25 E in T4 generation amongst the segregating population seems to be unique, showing complete protection against two different serious lepidopteran insect-pests without any yield penalty.

Interestingly, in spite of several modifications incorporated in *cry1A* genes and successful commercial cultivation of Bt-cotton, Bollgard I and II expressing Cry1Ac toxin and Cry1Ac + Cry2Ab toxins respectively, but obtaining transgenic plants expressing high-level of Cry1Ac is an exceptionally rare and random event (Perlak et al. 2001; Rawat et al. 2011). This is particularly due to some uncharacterized circumstances owing to the expression of Cry1Ac toxin that imparts negative effect and inhibits the *in vitro* regeneration and development of highly expressing transgenic events (Diehn et al. 1996; De Rocher et al. 1998). Recently, Cry1Ac toxin has been well-documented to exert detrimental effects on the regeneration of cotton and tobacco transgenics expressing the toxin protein above >0.002–0.005% of TSP (Rawat et al. 2011). The selection of Monsanto 531 event of transgenic Coker cotton, expressing high-level of Cry1Ac toxin from full-length, modified, synthetic *cry1Ac* like gene, is perhaps a chance phenomenon for possible introgression of the *cry1Ac* gene in the hot-spot region of cotton genome, resulting in very high expression of toxin, without any detrimental effect on the growth parameters (Perlak et al. 2001; Purcell et al. 2004; Zhang et al. 2008). Co-expression of two different Bt-*cry* genes, without affecting the overall growth either by direct gene transformation or introgression through conventional breeding is still not routine, except for few reports in recent years (Cao et al. 2008; Mehrotra et al. 2011).

The transgenic line Ab25 E of tomato upto T_6_ generation has consistently exhibited high expression of Cry1Ab toxin and complete protection against two different lepidopteran insects without affecting the growth and yield parameters. There is no evidence till date, of any *in vivo* toxicity of Cry1Ab on initial regeneration and development of plants transformed with *cry1Ab* gene (Kumar and Kumar 2004; Rawat et al. 2011). One instance can be recalled where the transgenic event Bt176 of Bt-*cry1Ab* maize was found to be detrimental against non-target pest monarch butterfly feeding on pollen of transgenic maize because *cry1Ab* gene was driven by pollen specific promoter (Sears et al. 2001). The cultivated tomato is strictly self-pollinated crop (cleistogamous nature of flower) and pollen specific promoter is not used for expression *cry1Ab* gene, therefore, the possibility of pollen-toxicity is ruled out in our case. Although, the use of single *cry1Ab* gene targeted against African maize stem borer in Bt-maize did not endure longer, and enforcement of high refugia and gene pyramiding strategy had prevented the development of resistance (Berg et al. 2013; Tabashnik et al. 2011).

Our data with large number of primary T_0_ transformants having single copy of the transgene showed typical Mendelian inheritance for single dominant trait with consistent improvement of Cry1Ab protein expression in subsequent generations, particularly in transgenic line Ab25.3 from T_2_ to T_4_ and results are similar to earlier reports (Fearing et al. 1997; Perlak et al. 2001). The best transgenic line Ab25 E in T_4_ generation amongst the five promising lines was selected, showing high expression of Cry1Ab toxin with single copy incorporation of modified *cry1Ab* gene as evident from Southern blot analyses of T_4_, T_5_ and T_6_ progenies of the transgenic plant.

Our results for over-expression of Cry1Ab in transgenic line Ab25 E showing effective toxicity against *S. litura* are similar to earlier reports of Bt-maize (Dutton et al. 2005; Berg et al. 2013). The high toxicity and strong efficacy against two different lepidopteran insects seems to reflect the synergistic interaction of high-level of Cry1Ab toxin with all possible available receptors or targets on the brush border membrane in both the insects. The results of receptor binding assay of midgut preparations with partially purified recombinant Bt-Cry1Ab toxin expressed in transgenic tomato plants corroborates well with our observed data for efficient protection against two different pests, as compared to earlier reports of toxicity to *H. armigera* only (Mandaokar et al. 2000; Kumar and Kumar 2004). Our results reflect the possible incorporation of the modified *cry1Ab* gene in the hot-spot locus of euchromatin region of the tomato genome for significant higher expression (Klee and Giovannoni 2011; The Tomato Genome Consortium, 2012), without affecting the growth parameters, similar to MON531 transgenic event of cotton (Perlak et al. 2001).

## Conclusions

To conclude the efficient insecticidal response of transgenic tomato line Ab25 E is due to the synergistic response of the over-expression of modified *cry1Ab* gene and efficient binding of Cry1Ab toxin with the different receptors and other possible target proteins located in the midgut of the two insects. This over expressing homozygous transgenic line can be deployed in tomato breeding programme for the introgression of insect-resistant trait in other commercially important varieties of tomato.

## Methods

### *Agrobacterium strain* and gene construct

*Agrobacterium tumefaciens* strain LBA4404 harbouring binary vector pBIN200 with modified and truncated 1845 bp *cry1Ab* gene (Sardana et al. 1996) driven by double enhancer *CaMV35S* promoter and neomycin phosphotransferase gene (*npt*II) for kanamycin resistance in pBIN 20 backbone (Hennegan and Danna 1998) has been used for tomato transformation as shown in Figure 1A. Cultures of *A. tumefaciens* were grown at 28°C in YEB medium containing 20 mg l^–1^ rifampicin, 50 mg l^–1^ kanamycin and 50 mg l^–1^ streptomycin for 24 h at 200 rpm and utilized for transformation of tomato leaf-discs.

### Plant material and explant preparation

Breeder seeds of tomato (*Solanum lycopersicum* L.) variety Pusa early dwarf (PED), obtained from National Seeds Corporation, New Delhi, India, were surface sterilized and germinated on semi-solid MS medium (Murashige and Skoog 1962), containing B5 vitamins (Gamborg et al. 1968), 3% (w/v) sucrose (HiMedia Labs, Mumbai, India) and 8 g l^–1^ agar (Sigma, USA) followed by incubation at 24 ± 2°C in dark and shifted after three days of 16:8 h light–dark cycle in culture room maintained at 22 ± 2°C, illuminated with light intensity of 100 μmol m^–2^ s^–1^ and 78 ± 4% relative humidity. Vegetative leaves from axenic tomato seedlings of 16–18 days, were excised and initially pre-cultured on MS medium supplemented with 2.5 mg l^–1^ 6-benzyladenine (BAP) + 0.5 mg l^–1^ indole-3-acetic acid (IAA) for three days prior to *Agrobacterium* co-cultivation (Koul et al. 2012).

### *Agrobacterium-mediated* transformation and plantlet regeneration

Tomato leaf discs were dipped in *Agrobacterium* suspension OD_600_ ≈ 0.25–0.3 (2 × 10^9^ cfu ml^–1^), in MS liquid co-cultivation medium supplemented with 100 μM acetosyringone (As) for 20 min. The leaf disc explants were dried on sterilized blotting paper and transferred onto co-cultivation medium comprising of MS salts + 3% (w/v) maltose + 100 μM As + 2.5 mg l^–1^ BAP + 0.5 mg l^–1^ IAA and co-cultivated in dark, for two days in the culture room. The explants thereafter were incubated on medium consisting of MS salts + 3% (w/v) maltose + 500 mg l^–1^ cefotaxime + 2.5 mg l^–1^ BAP + 0.5 mg l^–1^ IAA for 5–7 days and then transferred to shoot induction medium one (SIM-1) containing MS salts + 3% (w/v) maltose + 2.5 mg l^–1^ BAP + 0.5 mg l^–1^ IAA + 250 mg l^–1^ cefotaxime + 50 mg l^–1^ kanamycin and incubated for 21 days for the first screening of putative transgenic plants (I^st^ selection). The independent regenerated shoots with a pair of vegetative leaves developed on first cycle of kanamycin screening were identified and their first pair of vegetative leaves were excised , sub-cultured on the shoot induction medium supplemented with kanamycin (SIM–2) for successive second screening and selection of transgenic shoots. The independent shoots that regenerated after successive II^nd^ selection cycles were sub-cultured on shoot elongation medium (SEM) containing MS medium + 1.0 mg l^–1^ gibberellic acid (GA_3_) + 3% (w/v) sucrose and 50 mg l^–1^ kanamycin. The shoots recovered from SEM medium having 2–3 leaves were transferred to root induction medium (RIM) containing half-strength MS medium + 0.5 mg l^-1^ indole-3-butyric acid (IBA) + 50 mg l^–1^ kanamycin + 2% (w/v) sucrose and 0.8% (w/v) agar for 14 days. The rooted plantlets were transferred to plastic pots containing sterilized soilrite (Keltech Energies Ltd. Bengaluru, India) and irrigated with half-strength liquid MS medium devoid of sucrose. The pots were kept in a plant growth chamber (Conviron Adaptis 1000 PG, Canada) set at desired relative humidity starting from 90 to 70% for 14 days of hardening. The hardened plantlets were potted in earthen pots filled with soil: sand: farmyard manure (in 3:1:1 ratio) and transferred to glasshouse maintained at 24 ± 1°C under natural light for normal growth, development, flowering and seed setting.

Selfed seeds of the primary T_0_ transformants and transgenic plants were germinated on solidified half-strength MS medium supplemented with 50 mgl^-1^ kanamycin for segregation and selection of transgenic progeny.

### PCR and Southern blot analysis

Genomic DNA from leaves of primary T_0_ and transgenic plants was isolated using GenElute plant genomic DNA miniprep kit, according to the manufacturer’s instructions (Sigma, USA). PCR amplification of *cry1Ab* and *npt*II genes from plant genomic DNA (100 ng) was achieved by using set of primers amplifying 678 bp and 800 bp amplicon of *npt*II and *cry1Ab* genes respectively, in the GeneAmp® PCR system 9700 (PE Biosystems, USA). The set of primers for *npt*II gene was forward 5*′*-TATTCGGCTATGACTTGGGC-3*′* and reverse 5*′*-GCGAACGCTATGTCCTGATA-3*′*, while for *cry1Ab* gene forward primer was 5*′*-TGGTACAACACTGGCTTGGA-3*′* and reverse 5*′*-ATGGGATTTGGGTGATTTGA-3*′*. Southern blot hybridization was performed to confirm the integration of T-DNA into the genome of the transgenic plants according to Sambrook et al. (1989). Aliquot of 10 μg genomic DNA purified from the transgenic plants (T_0_–T_6_ generation) was digested overnight with *Eco*RI, cutting at single site within the T-DNA followed by gel electrophoresis and transferring onto BioBond Plus nylon membrane (Sigma, USA). The blot was hybridized at 58°C for 24 h with 1845 bp fragment of *cry1Ab* gene radio labeled with αP^32^dCTP (BRIT, Mumbai India) and washed under stringent conditions, exposed to Fuji screen for 48 h followed by scanning and documentation on Typhoon Trio Plus phosphoimager (GE Healthcare Life Sciences AB, Sweden).

### RT-PCR and quantitative real-time quantitative PCR (qPCR)

RT-PCR analysis of transgenic plants was done by synthesis of first-strand of cDNA with enhanced Avian RT-PCR kit using 5 μg of total RNA purified from the transgenic plant according to manufacturer’s instructions (Sigma, USA). The relative quantity of *cry1Ab* transcripts in transgenic tomato plants was analyzed by quantitative PCR performed in StepOne real-time PCR system (Applied Biosystems, USA) using Quantifast SYBR green PCR kit (Qiagen, Germany). Tomato *β*-*actin* gene (GenBank accession no. U60482) was used as endogenous control in all real-time PCR assays. The nucleotide sequences of the set of forward and reverse primers for *cry1Ab* gene were 5*′*-AAGGATTCTCCCACAGGTTG-3*′* and 5*′*-ATGGGATTTGGGTGATTTGAG-3*′* respectively, while for *β*-*actin* gene the forward 5*′*-GCTGGATTTGCTGGAGATGATGA-3*′* and reverse 5*′*-TCCATGTCATCCCAATTGCTAAC-3*′* giving an amplicon of 157 and 194 bp respectively. Total RNA extracted from 100 mg of leaf tissues was reverse transcribed into cDNA and used as template in real-time PCR assays with *cry1Ab* and *β*-*actin* gene-specific primers. Reverse transcription reaction was performed at 50°C for 10 min with initial denaturation at 95°C for 5 min (for activation of Hot-start Taq polymerase) followed by 40 amplification cycles comprising of 10 s denaturation at 95°C and combined annealing and extension for 30 s at 60°C in 25 μl reaction mixture, according to manufacturer’s instructions (Qiagen, Germany). The relative values obtained from the quantitation of mRNA were expressed as 2^-ΔΔCt^ where ΔCt represents the difference between Ct (cycle threshold) values of a target and the endogenous control (*β*-*actin*) in the same sample and ΔΔCt is the difference between the ΔCt value of a particular sample and that of the reference sample. The quantitative data of real-time PCR represent mean values with standard error of three independent experiments with three replicates of the transgenic plant.

### Quantitative estimation of recombinant Cry1Ab toxin

Vegetative leaves or other plant parts from 12 weeks old transgenic tomato plants of T_0_–T_6_ generations were used for protein extraction by grinding in 1:10 (w/v) ratio of plant tissue to PBST buffer (pH 7.4), in liquid nitrogen. The total soluble protein (TSP) concentration in cell-free extracts was determined by Bradford dye-binding procedure with bovine serum albumin (BSA) as standard protein (Bio-Rad, USA). The quantitative estimation of expressed recombinant Cry1Ab toxin in cell-free extracts of transgenic plants was determined by DAS-ELISA, using peroxidase labeled PathoScreen kit for Bt-Cry1Ab/1Ac protein (Agdia, USA). Cell-free extracts from leaves of transgenic plants were dispensed into wells of ELISA plate, pre-coated with primary antibody followed by reaction with secondary antibody conjugated with alkaline phosphatase to develop colour and detection of Cry1Ab toxin was monitored at 650 nm using SpectraMax 340PC spectrophotometer (Molecular Devices, USA). Expression levels were quantified on a linear standard curve plotted with pure Bt-Cry1Ab protein (Agdia, USA).

### Western immunoassay

Western immunoassay was performed with cell-free extracts of transgenic tomato plants expressing Cry1Ab toxin as described earlier (Sambrook et al. 1989). Aliquots of the total plant protein samples were boiled for 10 min with 2× sample loading dye [50 mM Tris–HCl (pH 6.8), 100 mM DTT, 2% SDS, 0.1% bromophenol blue and 10% glycerol] and electrophoresed on 10% denaturing SDS-PAGE. The resolved protein bands on SDS-PAGE were transferred onto immunoblot™ PVDF membrane (Bio-Rad, USA) using SD semi-dry transfer cell (Bio-Rad, USA) in transfer buffer [25 mM Tris base, 192 mM glycine (pH 8.3) and 0.1% SDS]. The membranes were blocked for 2 h at 25°C in blocking buffer and incubated with primary antibody (rabbit polyclonal to *B. thuringiensis* Cry1Ab toxin protein, Agdia, USA) diluted to 1:1000 ratio in blocking buffer, for 2 h at 25°C. The membranes were washed four times with PBST, followed by incubation with secondary antibody (goat polyclonal to rabbit IgG-alkaline phosphatase conjugated antibody, Sigma, USA) at 1:5000 dilution for 2 h at 25°C and colour development with BCIP-NBT substrate solution (Sigma, USA).

### Insect bioassay

The larval population of *H. armigera* and *S. litura* were reared in the insectary on an artificial diet (Gupta et al. 2004) at 26 ± 2°C, 70% relative humidity and 14 h light/ 10 h dark regime. The detached leaves from fourth to sixth nodes of untransformed control and transgenic plants of T_0_ to T_6_ generations were washed thoroughly with distilled water, blotted dry and placed in a plastic container with 10 number of second instar larvae of *H. armigera* or *S. litura* per leaf, in two replicates. Feeding was allowed for 48–96 h and the data on larval weight and percent mortality were analyzed statistically and results were co-related to the quantitative expression of Cry1Ab toxin. The green leaves and mature fruits of selected transgenic lines in T_4_ generation grown in glasshouse were also used for insect bioassay with second instar larvae of *H. armigera* and *S.litura*, respectively. Percent damage to untransformed control and transgenic leaf or fruit was considered as a function of loss in leaf area and fruit weight due to insect feeding. The leaf lamina was traced on the Whatman 3 MM paper, cut and weighed. The damaged portion of the leaf after insect bioassay was traced on the previously cut paper leaf and carefully removed and the damaged paper leaf was again weighed. Leaf area was calculated by dividing the weight of paper leaf by the weight of one cm^–2^ Whatman 3 MM paper. The leaf area after insect feeding was calculated similarly and finally the percent loss in leaf area was calculated by the formula (initial area – area after insect feeding) / initial area × 100. Similarly, the percent damage to fruits was calculated by the formula (initial weight – weight after insect feeding) / initial weight × 100. The leaf bioassay was performed thrice with five replicates, while the fruit bioassay was performed once with five replicates and the results were statistically analyzed by Student’s *t*-test.

### Separation of brush border membrane vesicles (BBMV) from the midgut of *H. armigera* and *S. litura*

BBMV were prepared by the differential magnesium-precipitation method of Upadhyay and Singh (2011). Fifth instar larvae were anaesthetized by placing them on ice for 5 min and dissected in MET buffer [300 mM Mannitol; 5 mM EGTA; 17 mM Tris.HCl, (pH 7.4)]. Midguts were excised from the insects, ground under liquid nitrogen and homogenized in 1:9 (w/v) MET buffer. Equal volume of 24 mM MgCl_2_ was added, mixed, kept on ice for 30 min, followed by centrifugation (2500 × g, 4°C, 15 min). The pellet was discarded and the supernatant was centrifuged (30, 000 × g, 4°C, 30 min). The pellet was re-suspended in half of the original volume of MET buffer and MgCl_2_ and the above steps were repeated. The final pellet was solubilized in phosphate buffered saline (PBS) containing 0.1% Tween-20, by overnight incubation at 4°C with gentle shaking. Protein content of BBMV suspension was measured by Bradford dye-binding assay. The BBMV preparations were used for *in vitro* interaction studies with partially purified recombinant Cry1Ab toxin isolated from the leaves of transgenic tomato plants.

### Receptor binding assay

Direct antigen coating enzyme-linked immunosorbent assay (DAC-ELISA) was performed for investigating the binding of recombinant Cry1Ab toxin with partially purified BBMV proteins isolated from fifth instar larvae of *H. armigera* and *S. litura*. The BBMV suspensions were serially diluted between 2.500 μg–0.040 μg per 100 μl bicarbonate buffer (pH 9.6), coated onto the 96-well ELISA plates and incubated overnight at 4°C (Greiner Bio-One, Germany). The ELISA plates were washed with PBST, blocked with 1% BSA in PBS containing 0.05% (w/v) Tween 20, washed and incubated with known concentration of cell-free leaf extracts of transgenic line Ab25 E and incubated at 37°C for 2 h followed by washing with PBST. The plates were first incubated with Cry1Ab primary antibody (Agdia, USA) for 2 h, followed by washing and incubation for 2 h with peroxidase-conjugated secondary antibody (Sigma, USA). After washing the ELISA plate with PBST, colour was developed by the addition of 200 μl of freshly prepared horseradish peroxidase (HRP) substrate solution (Merck, India) per well and absorbance was monitored at 490 nm. The experiment was repeated three times with three replicates.

### Statistical analysis

Each experiment was performed with five replicates, unless otherwise mentioned and repeated at least three times. Tissue culture data was subjected to analysis of variance by One-Way ANOVA (Gomez and Gomez 1984) to detect the significance of differences among treatment means using Duncan’s Multiple Range Test at *P* < 0.05. T_1_ seeds were germinated on MS basal medium supplemented with 50 mg l^-1^ kanamycin and subjected to χ^2^ fitness test for progeny segregation to compare the expected and observed data. All graphs were prepared using Sigma Plot software (Sigma Plot, USA).

## Electronic supplementary material

Additional file 1: Table S1: Segregation analysis of T_1_ transgenic tomato seeds developed with vector pBIN200 and the corresponding insect mortality data of T_0_ transformants. (DOC 67 KB)

Additional file 2: Figure S1: Comparative real-time PCR analysis of *cry1Ab* transcript in T0 plants showing fold change in expression with respect to the low expressing transgenic plant Ab2. Control: non-transformed plant. (PPT 110 KB)

Additional file 3: Figure S2: Average Bt-Cry1Ab protein (*striped bar*) in different parts of T0 transgenic tomato plant Ab25. (PPT 110 KB)

Additional file 4: Table S2: Detached leaf and fruit bioassay with T_4_ transgenic plants expressing Bt-Cry1Ab toxin. (DOC 36 KB)

Additional file 5: Figure S3: Control and T4 progeny of transgenic lines Ab25 C, Ab25 B, Ab25 A, Ab25 D and Ab25 E subjected to feeding assay by *S. litura*. Leaf area damage was calculated by the cut paper method. (PPT 802 KB)

Additional file 6: Figure S4: Control and T4 progeny of transgenic lines of Ab25 C, Ab25 B, Ab25 A, Ab25 D and Ab25 E subjected to feeding assay by *H. armigera*. Fruit damage was calculated by fruit weight method. (PPT 2 MB)

## Abbreviations

BAP: 6-Benzyladenine
BBMV: Brush border membrane vesicles
Cry: Crystal protein
DECaMV35S: *CaMV35S* promoter with duplicated enhancer
IAA: Indole-3-acetic acid
IBA: Indole-3-butyric acid
MS: Murashige and Skoog basal medium
nptII: Neomycin phosphotransferase
PBST: Phosphate buffered saline with Tween-20
TSP: Total soluble protein.
