# Supplementary material for: Transgenic tomato line expressing modified Bacillus thuringiensis cry1Ab gene showing complete resistance to two lepidopteran pests
Source: Springerplus. 2014 Feb 12;3:84. doi: 10.1186/2193-1801-3-84 (PMC3937457; doi:10.1186/2193-1801-3-84)
Supplement: Supplementary file 1 — Additional file 1: Table S1: Segregation analysis of T1 transgenic tomato seeds developed with vector pBIN200 and the corresponding insect mortality data of T0 transformants. (DOC 67 KB) [file 40064_2013_841_MOESM1_ESM.doc]

**Supplementary Table 1 Segregation analysis of T_1_ transgenic tomato seeds developed with vector pBIN200 and the corresponding insect mortality data of T_0_ transformants.**

| **T_0_ transgenic plants** | **Cry1Ab toxin**  **(% TSP)** | **% Weight loss** | | **% Mortality** | | **Response of seeds germinated on kanamycin selection medium** | | |
| --- | --- | --- | --- | --- | --- | --- | --- | --- |
|  |  | ***H.armigera*** | ***S.litura*** | ***H.armigera***  **(± 10)** | ***S.litura***  **(± 10)** | **Total**  **seeds** | **Kan^r^ / Kan^s^** | **χ^2^ value^a^** |
| Ab 1 | 0.04 | 61 ± 0.05 | 41 ± 0.06 | 80 | 50 | 24 | 16/8 | 0.89 |
| Ab 2 | 0.02 | 55 ± 0.21 | 18 ± 0.05 | 50 | 20 | 32 | 20/12 | 2.66 |
| Ab 3 | 0.03 | 77 ± 0.02 | 20 ± 0.05 | 80 | 30 | 76 | 64/12 | 3.44 |
| Ab 4 | 0.04 | 83.7 ± 0.13 | 33 ± 0.11 | 90 | 40 | 50 | 43/7 | 3.22 |
| Ab 5 | 0.034 | 78.6 ± 0.06 | 28 ± 0.01 | 80 | 30 | 14 | 10/4 | 0.10 |
| Ab 6 | 0.03 | 80.1 ± 0.05 | 22.1 ± 0.02 | 80 | 30 | 30 | 18/12 | 1.17 |
| Ab 7 | 0.05 | 88.7 ± 0.21 | 42 ± 0.02 | 90 | 50 | 32 | 24/8 | 0.00 |
| Ab 8 | 0.06 | 86.4 ± 0.11 | 31 ± 0.11 | 90 | 50 | 81 | 56/25 | 1.49 |
| Ab 9 | 0.025 | 46 ± 0.12 | 82 ± 0.21 | 50 | 30 | 64 | 47/17 | 0.08 |
| Ab 10 | 0.085 | * | 77 ± 0.05 | 100 | 80 | 57 | 42/15 | 0.05 |
| Ab 11 | 0.051 | 88.9 ± 0.09 | 52 ± 0.02 | 90 | 60 | 24 | 20/4 | 0.45 |
| Ab 12 | 0.052 | * | 48.2 ± 0.08 | 100 | 60 | 48 | 41/7 | 2.78 |
| Ab 13 | 0.075 | * | 67 ± 0.14 | 100 | 70 | 28 | 22/6 | 0.19 |
| Ab 14 | 0.03 | 67 ± 0.18 | 20 ± 0.11 | 80 | 30 | 50 | 41/9 | 1.31 |
| Ab 15 | 0.042 | 84 ± 0.08 | 46 ± 0.11 | 90 | 50 | 82 | 62/20 | 0.02 |
| Ab 16 | 0.05 | 88 ± 0.08 | 51 ± 0.24 | 90 | 60 | 17 | 13/4 | 0.02 |
| Ab 17 | 0.04 | 70 ± 0.18 | 34.04 ± 0.13 | 80 | 50 | 36 | 29/7 | 0.58 |
| Ab 18 | 0.026 | 42 ± 0.11 | 22 ± 0.05 | 50 | 30 | 78 | 61/17 | 0.43 |
| Ab 19 | 0.042 | 86.4 ± 0.11 | 40 ± 0.03 | 90 | 50 | 24 | 20/4 | 0.88 |
| Ab 20 | 0.035 | 82 ± 0.09 | 31 ± 0.06 | 80 | 40 | 42 | 34/8 | 0.79 |
| Ab 21 | 0.070 | * | 92.2 ± 0.10 | 100 | 90 | 68 | 50/18 | 0.08 |
| Ab 22 | 0.069 | 80 ± 0.22 | 56.04 ± 0.03 | 90 | 60 | 20 | 14/6 | 0.26 |
| Ab 23 | 0.020 | 56 ± 0.23 | 33 ± 0.15 | 50 | 20 | 32 | 22/10 | 0.66 |
| Ab 24 | 0.110 | * | 90.1 ± 0.08 | 100 | 90 | 43 | 32/11 | 0.01 |
| Ab 25 | 0.130 | * | * | 100 | 100 | 24 | 18/4 | 0.00 |

^a^χ^2^_1_ = 3.841 at ≤ 0.05

Kan^r^: Kanamycin-resistant, Kan^s^: Kanamycin-sensitive

***** 100 % mortality
