# Supplementary material for: Transgenic tomato line expressing modified Bacillus thuringiensis cry1Ab gene showing complete resistance to two lepidopteran pests
Source: Springerplus. 2014 Feb 12;3:84. doi: 10.1186/2193-1801-3-84 (PMC3937457; doi:10.1186/2193-1801-3-84)
Supplement: Supplementary file 2 — Additional file 2: Figure S1: Comparative real-time PCR analysis of cry1Ab transcript in T0 plants showing fold change in expression with respect to the low expressing transgenic plant Ab2. Control: non-transformed plant. (PPT 110 KB) [file 40064_2013_841_MOESM2_ESM.ppt]

## Slide 1
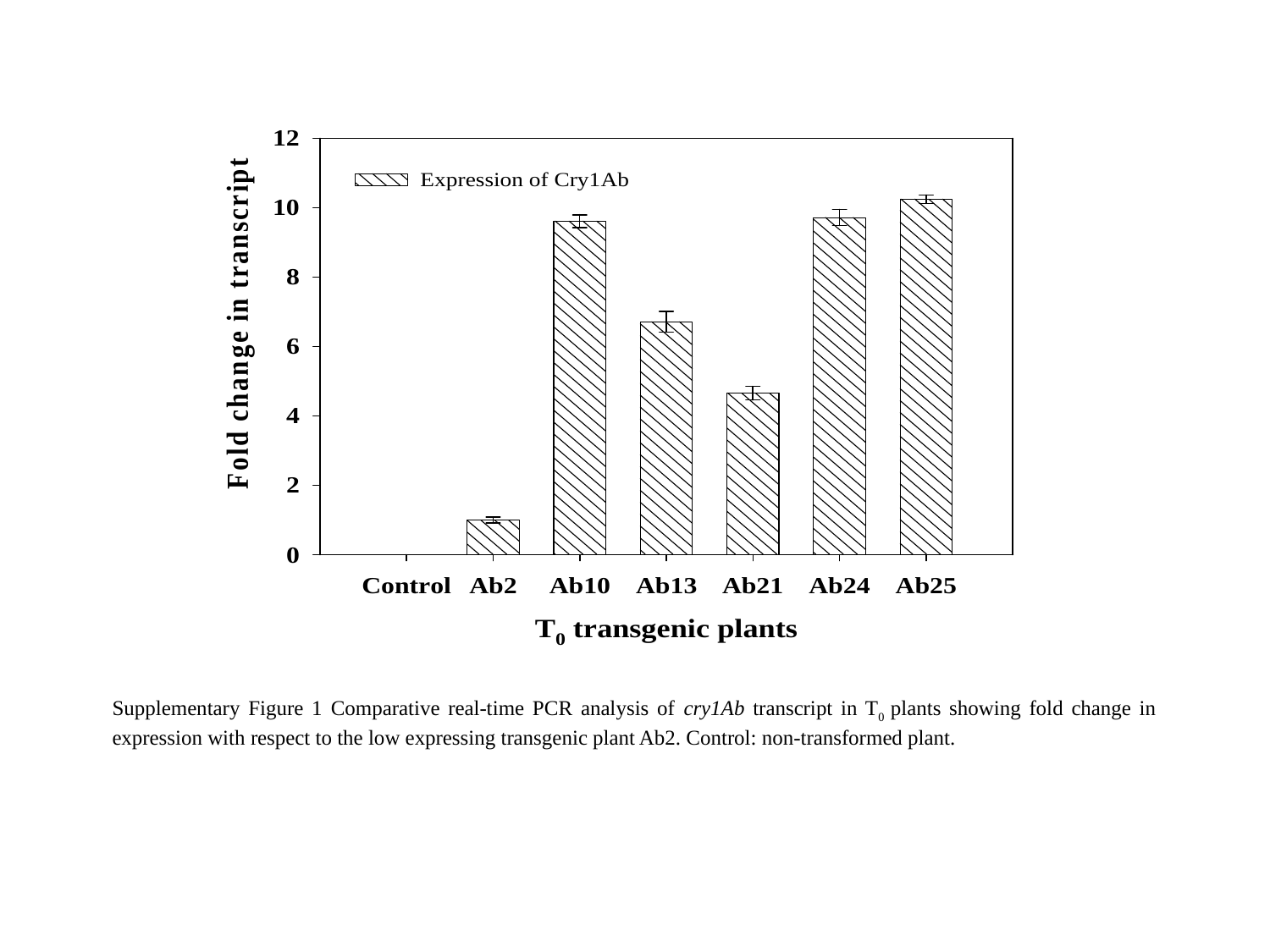

Supplementary Figure 1 Comparative real-time PCR analysis of cry1Ab transcript in T0 plants showing fold change in expression with respect to the low expressing transgenic plant Ab2. Control: non-transformed plant.
