# Supplementary material for: Transgenic tomato line expressing modified Bacillus thuringiensis cry1Ab gene showing complete resistance to two lepidopteran pests
Source: Springerplus. 2014 Feb 12;3:84. doi: 10.1186/2193-1801-3-84 (PMC3937457; doi:10.1186/2193-1801-3-84)
Supplement: Supplementary file 3 — Additional file 3: Figure S2: Average Bt-Cry1Ab protein (striped bar) in different parts of T0 transgenic tomato plant Ab25. (PPT 110 KB) [file 40064_2013_841_MOESM3_ESM.ppt]

## Slide 1
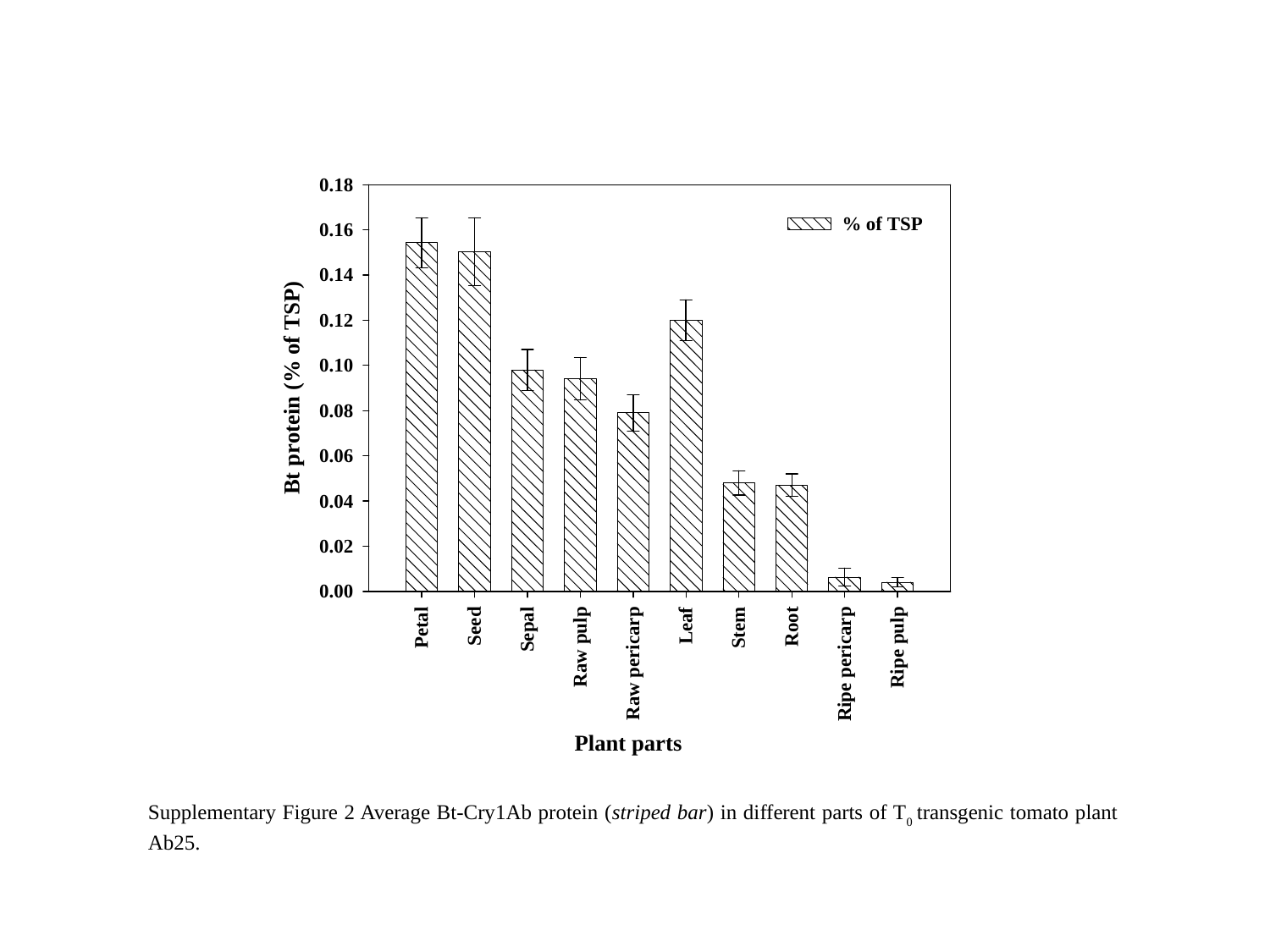

Supplementary Figure 2 Average Bt-Cry1Ab protein (striped bar) in different parts of T0 transgenic tomato plant Ab25.
