# Supplementary material for: Transgenic tomato line expressing modified Bacillus thuringiensis cry1Ab gene showing complete resistance to two lepidopteran pests
Source: Springerplus. 2014 Feb 12;3:84. doi: 10.1186/2193-1801-3-84 (PMC3937457; doi:10.1186/2193-1801-3-84)
Supplement: Supplementary file 4 — Additional file 4: Table S2: Detached leaf and fruit bioassay with T4 transgenic plants expressing Bt-Cry1Ab toxin. (DOC 36 KB) [file 40064_2013_841_MOESM4_ESM.doc]

| **Transgenic**  **lines** | **^a^Leaf area (cm^-2^)** | **Leaf area**  **after feeding by**  ***S. litura* (cm^-2^)** | **Loss of leaf area**  **(%)** | **^b^Fruit weight**  **(g)** | **Fruit weight**  **after feeding by *H. armigera* (g)** | **Loss in fruit weight (g)** | **Loss in fruit weight**  **(%)** |
| --- | --- | --- | --- | --- | --- | --- | --- |
| Control | 31.10 ± 6.91 | 22.95 ± 5.91 | 26.14 ± 3.91 | 43.33 ± 5.92 | 35.95 ± 5.99 | 7.39 ± 0.88 | 17.30 ± 3 .11 |
| Ab25 A | 18.29 ± 3.39 | 18.22 ± 3.38 | 0.39 ± 0.19 | 30.19 ± 2.18 | 29.2 ± 1.89 | 0.91 ± 0.59 | 2.96 ± 1.85 |
| Ab25 B | 21.57 ± 1.82 | 20.78 ± 1.12 | 3.48 ± 2.89 | 29.11 ± 4.38 | 28.01 ± 3.72 | 1.10 ± 0.68 | 3.54 ± 2.03 |
| Ab25 C | 20.39 ± 1.73 | 19.55 ± 1.52 | 3.75 ± 2.46 | 26.13 ± 3.54 | 24.86 ± 2.98 | 1.28 ± 0.84 | 4.70 ± 2.63 |
| Ab25 D | 22.40 ± 2.05 | 22.35 ± 2.07 | 0.25 ± 0.12 | 36.14 ± 4.40 | 35.11 ± 3.76 | 1.03 ± 0.77 | 2.72 ± 1.79 |
| Ab25 E | 23.08 ± 3.67 | 23.04 ± 3.69 | 0.20 ± 0.16 | 42.77 ± 5.38 | 41.96 ± 5.56 | 0.87 ± 0.23 | 1.71 ± 1.18 |

**Supplementary Table 2 Detached leaf and fruit bioassay with T_4_ transgenic plants expressing Bt-Cry1Ab toxin.**

^a^ For leaf bioassay three experiments were performed with five replicates from each line

^b^ For fruit bioassay single experiment was performed with five replicates from each line
