# Supplementary material for: Transgenic tomato line expressing modified Bacillus thuringiensis cry1Ab gene showing complete resistance to two lepidopteran pests
Source: Springerplus. 2014 Feb 12;3:84. doi: 10.1186/2193-1801-3-84 (PMC3937457; doi:10.1186/2193-1801-3-84)
Supplement: Supplementary file 5 — Additional file 5: Figure S3: Control and T4 progeny of transgenic lines Ab25 C, Ab25 B, Ab25 A, Ab25 D and Ab25 E subjected to feeding assay by S. litura. Leaf area damage was calculated by the cut paper method. (PPT 802 KB) [file 40064_2013_841_MOESM5_ESM.ppt]

## Slide 1
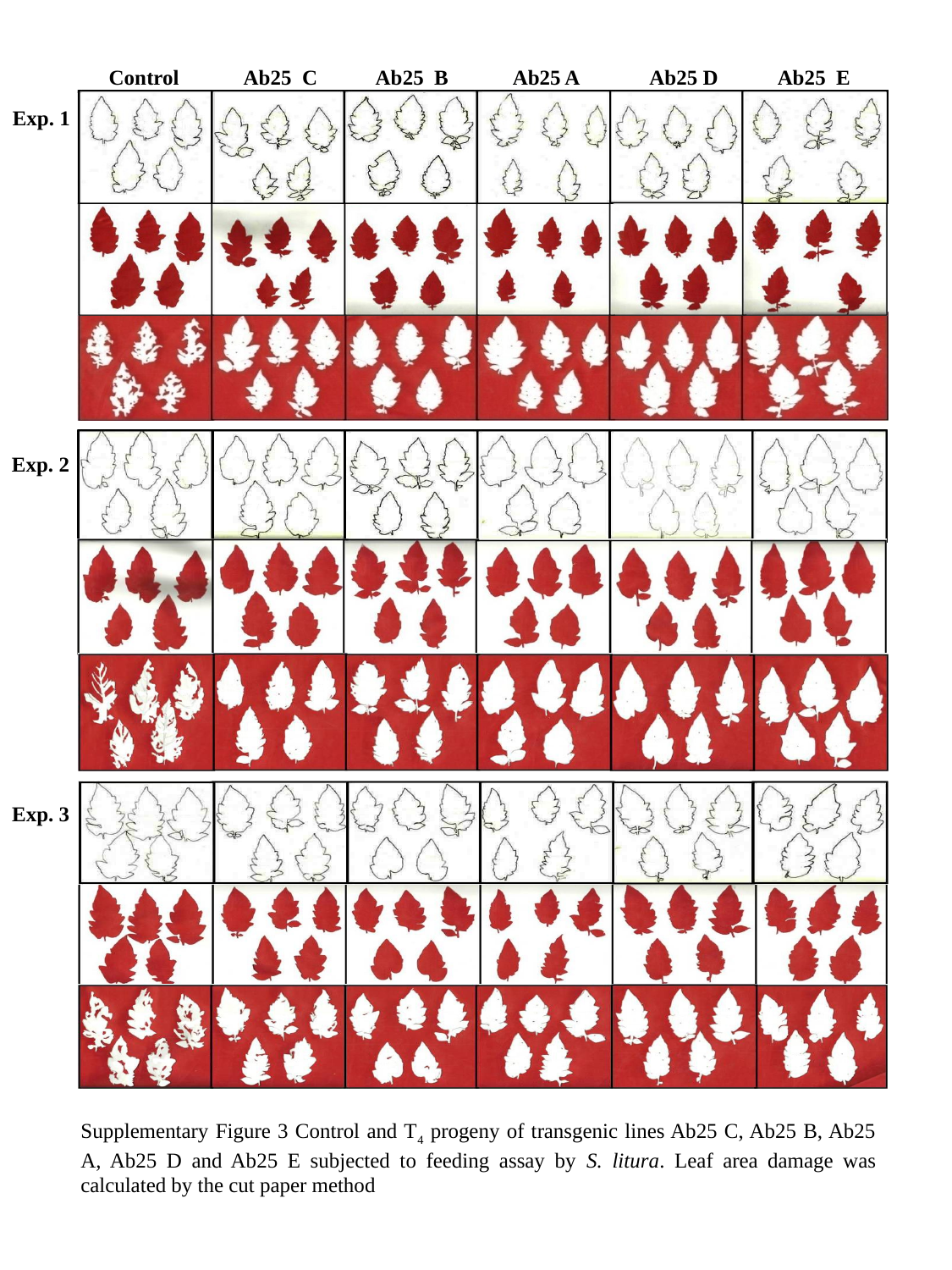

Control
Ab25 C
Ab25 B
Ab25 A
Ab25 D
Ab25 E
Exp. 1
Exp. 2
Exp. 3
Supplementary Figure 3 Control and T4 progeny of transgenic lines Ab25 C, Ab25 B, Ab25 A, Ab25 D and Ab25 E subjected to feeding assay by S. litura. Leaf area damage was calculated by the cut paper method
