# Supplementary material for: Transgenic tomato line expressing modified Bacillus thuringiensis cry1Ab gene showing complete resistance to two lepidopteran pests
Source: Springerplus. 2014 Feb 12;3:84. doi: 10.1186/2193-1801-3-84 (PMC3937457; doi:10.1186/2193-1801-3-84)
Supplement: Supplementary file 6 — Additional file 6: Figure S4: Control and T4 progeny of transgenic lines of Ab25 C, Ab25 B, Ab25 A, Ab25 D and Ab25 E subjected to feeding assay by H. armigera. Fruit damage was calculated by fruit weight method. (PPT 2 MB) [file 40064_2013_841_MOESM6_ESM.ppt]

## Slide 1
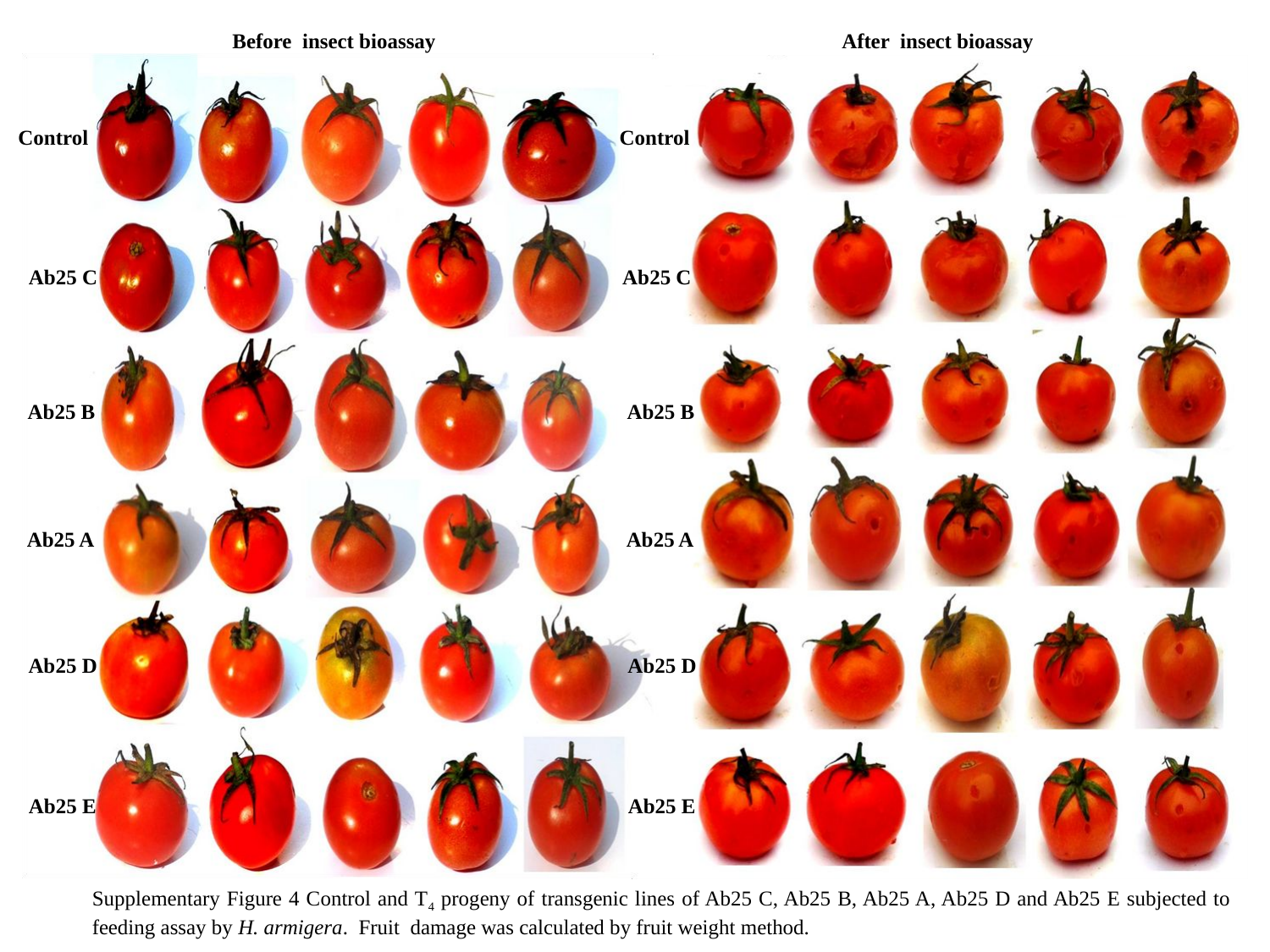

Before insect bioassay
After insect bioassay
Control
Ab25 C
Ab25 B
Ab25 A
Ab25 D
Ab25 E
Control
Ab25 C
Ab25 B
Ab25 A
Ab25 D
Ab25 E
Supplementary Figure 4 Control and T4 progeny of transgenic lines of Ab25 C, Ab25 B, Ab25 A, Ab25 D and Ab25 E subjected to feeding assay by H. armigera. Fruit damage was calculated by fruit weight method.
